# Evolving Trends and Perioperative Outcomes of Surgical Treatment for Male Stress Urinary Incontinence: Results from the GRAND Study Register

**DOI:** 10.1016/j.euros.2026.06.001

**Published:** 2026-06-22

**Authors:** Yannic Volz, Marc Kidess, Julian Hermans, Patrick Keller, Ricarda Bauer, Christian Gozzi, Michael Chaloupka, Julian Marcon, Philipp Weinhold, Christian Stief, Gerald Bastian Schulz, Nikolaos Pyrgidis

**Affiliations:** a Department of Urology, LMU University Hospital, Munich, Germany; b Urologische Klinik Planegg, Munich, Germany; c City Klinik Bozen, Bolzano, Italy

**Keywords:** Artificial urinary sphincter, Sling, Stress urinary incontinence, Perioperative outcomes

## Abstract

**Background and objective:**

Stress urinary incontinence (SUI) remains a debilitating complication after treatment for prostate cancer or benign prostatic obstruction. While artificial urinary sphincter (AUS) implantation is the gold standard, sling procedures are widely adopted for selected patients. We aimed to analyze long-term trends, perioperative outcomes, and explantation patterns of AUS and sling procedures for male SUI in Germany.

**Methods:**

We performed a population-based study using the German Nationwide Inpatient Sample (GRAND) from 2005 to 2023. Men undergoing AUS or sling implantation were identified through procedure codes. Primary outcomes were in-hospital morbidity, mortality, and length of stay (LOS). Multivariable regression models were adjusted for age, comorbidities, prior radiotherapy, and year of surgery. Reasons for explantation and reimplantation were also assessed.

**Key findings and limitations:**

A total of 24 234 men underwent SUI surgery (AUS: 63%, nonadjustable sling: 32%, and adjustable sling: 4.7%). Median age was 72 yr. AUS implantation remained most frequent, although it declined slightly in recent years. Use of nonadjustable sling peaked in 2012 but decreased thereafter, whereas use of adjustable sling procedures increased steadily. Procedure volumes markedly decreased during COVID-19. Perioperative mortality was <0.1% across groups. Nonadjustable slings were associated with a higher risk of acute urinary retention (odds ratio [OR]: 1.1, *p* = 0.020) but with a lower risk of wound infection (OR: 0.5, *p* < 0.001) than AUS. No statistically significant differences were observed between adjustable slings and AUS in terms of perioperative morbidity. LOS was longer in AUS (median 6 d) than in slings (median 5 d; *p* < 0.001). Two-cuff AUS were associated with longer LOS than single-cuff devices. Explantations occurred most often due to infection or mechanical failure for AUS, and for different reasons for slings. Limitations include reliance on administrative coding without functional or long-term patient-reported outcomes.

**Conclusions and clinical implications:**

Male SUI surgery is safe, with AUS remaining the most frequent procedure in Germany. Adjustable slings represent an expanding option for selected patients.

ADVANCING PRACTICE **What does this study add?**

This nationwide, population-based analysis provides the most comprehensive contemporary data on male stress urinary incontinence (SUI) surgery. We demonstrate that AUS remains the predominant treatment, although sling procedures—particularly adjustable systems—are increasingly utilized. Our findings highlight very low perioperative morbidity and mortality across all modalities, but distinct differences in complication and explantation patterns. Importantly, the overall number of procedures suggests persistent undertreatment of male SUI, underscoring the need for improved awareness and access to care.

**Clinical Relevance**

This study confirms that all three surgical options for male SUI — AUS, non-adjustable slings, and adjustable slings — are safe, with distinct perioperative profiles that can guide patient counseling and shared decision-making. Non-adjustable slings carry a modestly higher risk of acute urinary retention but a lower risk of wound infection compared to AUS, while adjustable slings show a favorable safety profile with growing utilization. Within the AUS cohort, two-cuff devices are associated with longer hospitalization and higher rates of urinary retention, likely reflecting greater surgical complexity. Explantation data highlight infection and mechanical failure as the primary challenges for AUS durability, whereas sling explantations are more heterogeneous and frequently reflect insufficient continence outcomes. The overall low procedure volumes relative to the prevalence of SUI underscore the need for improved awareness and access to care. Associate Editor: Véronique Phé.

**Patient Summary**

We studied men in Germany who underwent surgery for stress urinary incontinence between 2005 and 2023. The artificial urinary sphincter remains the most common operation, but sling procedures are increasingly used. All treatments are safe, but many men with incontinence still do not receive surgery.

## 1 Introduction

Stress urinary incontinence (SUI) remains among the most frequent and devastating complications following treatment for prostate cancer or benign prostatic obstruction (BPO), exerting a considerable negative influence on patients’ quality of life [1]. Radical prostatectomy has been associated with SUI rates of roughly 20% in prior reports [2], [3], and surgical treatment for BPO with SUI rates of up to 5.0% [4]. The latter highlights the ongoing need for effective surgical treatment modalities in patients with persistent SUI. In recognition of this, the European Association of Urology launched the public awareness initiative “An Urge to Act” [5]. Based on the previous notion, a plethora of therapeutic options have emerged over the last few years, with promising results for SUI and quality of life [6], [7].

Currently, the gold standard for treating severe SUI is the placement of an artificial urinary sphincter (AUS) [8], [9]. Despite AUS having been used for more than 50 yr, patients often wish for an electronic AUS [10]. Other treatment options, especially for milder forms of SUI, include fixed, nonadjustable slings such as the AdvanceXP sling, or adjustable slings such as the Adjustable Transobturator Male System [11]. Both sling types have demonstrated acceptable safety and efficacy, resulting in high patient satisfaction rates [12], [13]. Available epidemiological data from the USA indicate that the number of surgeries performed for the treatment of SUI has generally increased but has begun to stabilize in recent years [14].

Although male SUI is highly relevant, data on general outcomes and treatment trends in Europe are relatively sparse. Therefore, the current work aimed to analyze current developments in the surgical management of male SUI and to determine outcomes and complications using a large national inpatient database from Germany. As our work was descriptive in nature, we did not investigate the causal pathways between surgical management of male SUI and perioperative outcomes.

## 2 Methods

### 2.1 German nationwide inpatient data

The current study used data from the German Nationwide Inpatient Sample (GRAND) from 2005 to 2023. The database includes anonymized patient-level hospital records from all German hospitals (excluding psychiatric, forensic, and military) and is maintained by the Federal Statistical Office. Since the introduction of the German Diagnoses-Related Groups (G-DRG) system in 2004, all inpatient treatments have been systematically reported, with diagnoses and procedures coded using International Classification of Diseases 10th revision, German Modification (ICD-10-GM) and the German Operationen- und Prozedurenschlüssel (OPS). OPS is a standardized classification system maintained by the German Institute for Drugs and Medical Devices. In the GRAND registry, procedures are identified based on these OPS codes. The OPS serves as the national equivalent to procedure coding systems such as Current Procedural Terminology or ICD-10 Procedural Coding System (ICD-10-PCS) and is used for hospital reimbursement and registry documentation.

### 2.2 Study cohort and outcome measures

Males who underwent surgical management for SUI, identified by specific OPS codes for AUS implantation (OPS Code: 5-597.00 or 5-597.01), adjustable slings (OPS Code: 5-596.75), and nonadjustable slings (OPS Code: 5-598.0) were included in the study. Associated diagnoses and comorbidities such as obesity, hypertension, diabetes mellitus, chronic kidney disease, chronic heart failure, and a history of pelvic radiation were captured using ICD-10-GM codes. Only the most frequently coded comorbidities could be analyzed, as the database depends entirely on the documentation practices of the different clinics. The primary outcomes were in-hospital morbidity and mortality, as well as length of stay (LOS). We also evaluated trends in AUS, adjustable slings, and nonadjustable slings, as well as differences in patient characteristics.

### 2.3 Statistical analysis

All analyses were conducted from the Research Data Center of the Federal Statistical Office using R scripts provided by our research group. Since only aggregated output was provided to the investigators from the Research Data Center and no individual-level data were accessible, ethical review and patient consent were not required following the national data protection regulations. Associations between surgical intervention type and perioperative outcomes were assessed using multivariable logistic and linear regression models adjusted for age, hypertension, obesity, history of chronic kidney disease or heart failure, diabetes, prior radiation therapy, and year of surgery. To account for temporal changes in surgical practice, the year of surgery was entered as a categorical covariate using dummy coding. Owing to the low number of events for mortality and sepsis, multivariable regression analyses were not performed for these endpoints due to the substantial risk of overfitting; these outcomes are therefore presented descriptively only. Logistic regression models were expressed as odds ratios [OR] with 95% confidence intervals [CIs]. A *p* value <0.05 was considered statistically significant for all measures, and all statistics were reported based on the European Urology guidelines [15], [16].

## 3 Results

### 3.1 Baseline characteristics and trends

A total of 24 234 patients were identified who underwent implantation of a device for surgical treatment of SUI in Germany between 2005 and 2023. Among them, 15 271 (63%) received an AUS, 7819 (32%) a nonadjustable sling, and 1144 (4.7%) an adjustable sling. The median age of the cohort was 72 yr (interquartile range [IQR]: 67–76), and most patients were aged between 60 and 79 yr. Comorbid conditions were common, with 55% (*n* = 13 309) of the included patients having hypertension, 17% (*n* = 4161) diabetes, and 5.8% (*n* = 1401) chronic kidney disease. A history of pelvic radiation was documented in 11% (*n* = 2595) of cases. The baseline characteristics of the included males are presented in Table 1.

**Table 1:** Baseline characteristics of AUS, non-adjustable and adjustable slings for stress urinary incontinence

Characteristic	AUS (*n* = 15 271)	Non-adjustable sling (*n* = 7819)	Adjustable sling (*n* = 1144)
Age, Yrs, Median (IQR)	72 (67–76)	71 (66–75)	73 (68–77)
Clinical comorbidities, *n* (%)			
Obesity	1001 (6.6%)	369 (4.7%)	54 (4.7%)
Hypertension	8586 (56%)	4108 (53%)	615 (54%)
Diabetes	2808 (18%)	1154 (15%)	199 (17%)
Chronic kidney disease	1000 (6.5%)	339 (4.3%)	62 (5.4%)
Chronic heart failure	402 (2.6%)	140 (1.8%)	22 (1.9%)
Prior radiation therapy	2025 (13%)	467 (6.0%)	103 (9.0%)
Age group, *n* (%)			
<49	147 (0.3%)	30 (0.4%)	2 (0.2%)
50–59	711 (4.7%)	442 (5.6%)	48 (4.2%)
60–69	4217 (28%)	2213 (28%)	281 (25%)
70-79	383 (55%)	355 (56%)	610 (53%)
>80	1813 (12%)	779 (10%)	203 (18%)

AUS = artificial urinary sphincter; IQR = interquartile range.

**Fig. 1:**
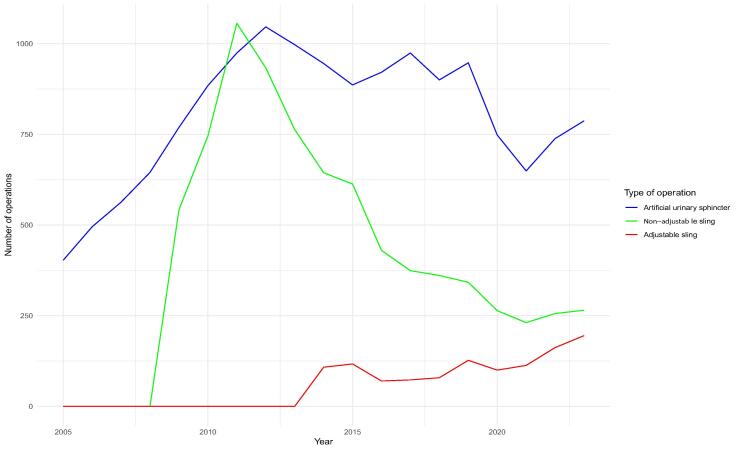
The annual trends of artificial urinary sphincter, non-adjustable, and adjustable slings for stress urinary incontinence in Germany.

In the AUS cohort, 11 770 patients (77%) received a single-cuff device, whereas 3501 (23%) underwent implantation of a two-cuff system. The baseline characteristics of patients receiving one- versus two-cuff AUS are displayed in Supplementary Table 1. Over the study period, AUS implantation remained the most common surgery, although its use has declined in recent year. Similarly, the use of nonadjustable slings has declined in recent year, with 1046 implantations in 2012 versus 787 in 2023. On the contrary, the use of adjustable slings is increasing yearly, with 195 performed in 2023. Notably, the surgical treatment of male SUI decreased substantially during the COVID-19 pandemic (Fig. 1).

### 3.2 AUS versus adjustable and nonadjustable slings

Perioperative mortality was extremely low across all the treatment groups. Specifically, 10 deaths (<0.1%) occurred in the AUS group and three (<0.1%) in the nonadjustable sling group. No deaths were recorded in the adjustable sling group. Nonadjustable slings were associated with a slightly increased risk of retention (12% vs 11%, OR: 1.1, 95% CI: 1.02–1.21, *p* = 0.020) but with a lower risk of in-hospital surgical wound infections (0.7% vs 1.4%, OR: 0.5, 95% CI: 0.4–0.7, *p* < 0.001) than AUS. Moreover, the median LOS was longer for AUS (6 d; IQR: 5–8), than for nonadjustable (5 d; IQR: 4–6) and adjustable slings (5 d; IQR: 3–6). In the multivariable analysis, nonadjustable sling implantation was associated with a significantly shorter LOS by 2.1 d (95% CI: 2.2–2.0, *p* < 0.001), and adjustable sling implantation by 0.98 d (95% CI: 1.2–0.7, *p* < 0.001) than AUS. All regression analyses are presented in Table 2.

**Table 2:** Multivariable linear and logistic regression analysis of AUS versus non-adjustable and adjustable slings for stress urinary incontinence on major perioperative outcomes

Outcome	AUS			Non-adjustable sling			Adjustable sling		
	Cases	Estimate OR (95% CI)	*p* value	Cases	Estimate OR (95% CI)	*p* value	Cases	Estimate OR (95% CI)	*p* value
Mortality	10 (<0.1%)	—	—	3 (<0.1%)	—	—	0 (0.0%)	—	—
Acute urinary retention	1606 (11%)	—	—	901 (12%)	1.1 (1.02–1.21)	0.020 a	94 (8.2%)	0.9 (0.8–1.2)	0.700
Length of hospital stay	6 (5–8)	—	—	5 (4–6)	−2.1 (−2.2 to −2)	<0.001 a	5 (3–6)	−0.98 (−1.2 to −0.7)	<0.001 a
Sepsis	16 (0.1%)	—	—	8 (0.1%)	—	—	0 (0.0%)	—	—
Surgical wound infection	221 (1.4%)	—	—	55 (0.7%)	0.5 (0.4–0.7)	<0.001 a	12 (1.0%)	1 (0.52–1.7)	0.900 a

CI = confidence interval; OR = odds ratio; AUS = artificial urinary sphincter.

Values are presented as *n* (%) or *n* (range) or OR (95% CI). All models are adjusted for age, hypertension, obesity, history of chronic kidney disease or heart failure, diabetes, prior radiation therapy, and year of surgery.

a Statistically significant *p* values.

### 3.3 One-cuff AUS versus two-cuff AUS

Two-cuff AUS were associated with a higher rate of acute urinary retention (12% vs 10%, OR: 1.13, 95% CI: 1.01–1.27, *p* = 0.040) and longer LOS by 0.6 d (95% CI: 0.4–0.8, *p* < 0.001) than one-cuff systems. No significant differences were observed for wound infections between the two AUS subtypes. All regression analyses are available in Table 3.

**Table 3:** Multivariable linear and logistic regression analysis of one- versus two-cuff AUS on major perioperative outcomes

Outcome	One-cuff AUS			Two-cuff AUS		
	Cases	Estimate: OR (95% CI)	*p* value	Cases	Estimate: OR (95% CI)	*p* value
Mortality	10 (<0.1%)	—	—	0 (0.0%)	—	—
Acute urinary retention	1183 (10%)	—	—	423 (12%)	1.13 (1.01–1.27)	0.047 a
Length of hospital stay	6 (5–8)	—	—	7 (6-9)	0.6 (0.4–0.8)	<0.001 a
Sepsis	12 (0.1%)	—	—	4 (0.1%)	—	—
Surgical wound infection	170 (1.4%)	—	—	51 (1.5%)	0.9 (0.7–1.2)	0.500

AUS = artificial urinary sphincter; CI = confidence interval; OR = odds ratio.

Values are presented as *n* (%) or OR (95% CI). All models are adjusted for age, hypertension, obesity, history of chronic kidney disease or heart failure, diabetes, prior radiation therapy, and year of surgery.

a Statistically significant *p* values.

### 3.4 Explantation or reimplantation

We identified 5700 patients who underwent explantation of an AUS in Germany between 2005 and 2023. Among them, the explantation was mandatory due to infection in 2708 (48%) cases, mechanical failure in 1840 (32%) cases, erosion of the urethra in 425 (7.5%) cases, and for other reasons in 727 (13%) cases. Accordingly, we identified 1435 patients who underwent explantation of an adjustable sling in Germany between 2005 and 2023. Among them, the explantation was mandatory due to infection in 421 (29%) cases, mechanical failure in 401 (28%) cases, erosion of the urethra in 58 (4.0%) cases, and for other reasons in 555 (39%) cases.

Moreover, 2629 patients underwent concomitant explantation with reimplantation of an AUS (1795 one-cuff and 834 two-cuff) in Germany between 2005 and 2023. In most cases (1215, 46%), concomitant explantation with reimplantation of an AUS was necessary due to mechanical failure. On the contrary, 2531 patients underwent concomitant explantation with reimplantation of an adjustable sling in Germany between 2005 and 2023. Only 147 (5.8%) cases were attributed to mechanical failure. The baseline characteristics of the included males are presented in Table 4, Table 5.

**Table 4:** Baseline characteristics of patients undergoing AUS explantation or explantation with concomitant reimplantation

Characteristic	Explantation of AUS (*n* = 5700)	Explantation with reimplantation of AUS (*n* = 2629)
Age, Yr, median (IQR)	75 (70–79)	74 (69–78)
Clinical comorbidities, *n* (%)		
Obesity	344 (6.0%)	155 (5.9%)
Hypertension	3475 (61%)	1514 (58%)
Diabetes	1272 (22%)	469 (18%)
Chronic kidney disease	746 (13%)	197 (7.5%)
Chronic heart failure	369 (6.5%)	68 (2.6%)
Prior radiation therapy	740 (13%)	226 (8.6%)
Age group, *n* (%)		
<49	133 (2.3%)	45 (1.7%)
50–59	152 (2.7%)	86 (3.3%)
60–69	950 (17%)	496 (19%)
70–79	2784 (49%)	1408 (54%)
>80	1681 (29%)	594 (23%)
Yr of operation, *n* (%)		
2005	130 (2.3%)	51 (1.9%)
2006	181 (3.2%)	72 (2.7%)
2007	162 (2.8%)	65 (2.5%)
2008	187 (3.3%)	80 (3.0%)
2009	223 (3.9%)	100 (3.8%)
2010	242 (4.2%)	131 (5.0%)
2011	266 (4.7%)	131 (5.0%)
2012	327 (5.7%)	189 (7.2%)
2013	328 (5.8%)	186 (7.1%)
2014	352 (6.2%)	270 (10.3%)
2015	354 (6.2%)	170 (6.5%)
2016	387 (6.8%)	142 (5.4%)
2017	346 (6.1%)	141 (5.4%)
2018	372 (6.5%)	148 (5.6%)
2019	438 (7.7%)	161 (6.1%)
2020	388 (6.8%)	148 (5.6%)
2021	298 (5.2%)	145 (5.5%)
2022	356 (6.2%)	141 (5.4%)
2023	363 (6.4%)	158 (6.0%)

AUS = artificial urinary sphincter; IQR = interquartile range.

**Table 5:** Baseline characteristics of patients undergoing adjustable sling explantation or explantation with concomitant reimplantation

Characteristic	Explantation of Adjustable sling (*n* = 1435)	Explantation with reimplantation of Adjustable sling (*n* = 2531)
Age, Yrs, median (IQR)	73 (69–78)	73 (69–77)
Clinical comorbidities, *n* (%)		
Obesity	80 (5.6%)	145 (5.7%)
Hypertension	796 (55%)	1,415 (56%)
Diabetes	257 (18%)	402 (16%)
Chronic kidney disease	122 (8.5%)	124 (4.9%)
Chronic heart failure	50 (3.5%)	51 (2.0%)
Prior radiation therapy	123 (8.6%)	225 (8.9%)

IQR = interquartile range.

## 4 Discussion

Male SUI remains a common challenge in contemporary urological practice. However, evidence on its incidence, available treatment modalities, and safety remains limited. To date, only one study has reported data on current trends in surgical treatment of SUI, without addressing safety aspects or complications [17]. Moreover, no data is available from the COVID-19 period, underscoring the need for more recent analyses to capture current trends and developments. The present study, therefore, aimed to provide a holistic approach to the trends, safety, and in-hospital complication outcomes of surgeries for male SUI.

In this nationwide, population-based analysis spanning nearly 2 decades, we suggest that AUS implantation remains the predominant surgical approach in Germany for male SUI, with low in-hospital morbidity and mortality. Compared with AUS, both adjustable and nonadjustable sling procedures were associated with shorter LOS and comparable safety profiles, although nonadjustable slings were linked to a slightly increased risk of acute urinary retention, but with better outcomes in terms of in-hospital surgical wound infections. Notably, complication rates across all treatment modalities were low. Furthermore, within the AUS cohort, implantation of a two-cuff device resulted in prolonged LOS and a higher risk of postoperative urinary retention compared with single-cuff systems. Yet, this may be explained by the fact that more complex surgical scenarios and more extensive preexisting conditions, or even revision surgery, often necessitate implanting a two-cuff system [18]. Overall, LOS appears to be longer than in data from the USA, where early discharge, including same-day surgery, is frequently practiced [19]. In Germany, however, this is largely determined by the DRG system, which requires a minimum LOS to ensure reimbursement of surgical costs by health insurance agencies.

Our findings on trends in male SUI are in line with both epidemiological data from the USA [14] and the multinational SATURN registry [7], highlighting a general trend toward diversifying surgical strategies for SUI. A similar observation was reported by Baunacke et al, who demonstrated an overall decline in male SUI surgery despite the increasing number of radical prostatectomies [17]. This discrepancy suggests an undertreatment of male SUI, leaving many patients with a persistently reduced quality of life. Consequently, this aligns with the recent findings of Shaw et al, who reported that only 12% of patients with SUI in the USA receive treatment [20]. Interestingly, our findings demonstrated an increase in the use of nonadjustable slings in 2011, which could be attributed to the introduction of novel slings such as the AdVanceXP at that time. The introduction of the next-generation AdvanceXP sling might have made surgeons more inclined to perform sling implantation. In addition, several publications appearing around 2010 demonstrated favorable outcomes, which likely increased interest among both urologists and patients [21], [22]. Accordingly, the cases of nonadjustable slings have decreased in recent years, which may be explained by Food and Drug Administration mesh ban [23], and by accumulating evidence suggesting that prior radiotherapy may compromise the success rates of nonadjustable slings [21], [24]. It should also be noted that the observed better outcomes after AUS versus nonadjustable slings might be a temporal effect, since nonadjustable sling implantation was mostly performed during the early period of the present cohort.

Importantly, we observed a substantial reduction in all surgical procedures for male SUI during the COVID-19 pandemic. This finding is consistent with previous reports of reductions in elective urologic procedures during the pandemic, largely attributable to healthcare resource reallocation, restrictions on elective surgery, and patient-related delays in seeking care [25]. Although procedure volumes partially recovered in subsequent years, the transient reduction may have contributed to a backlog of untreated patients and could influence future demand for SUI surgery.

Perioperative safety outcomes were reassuring across all modalities. As expected, in-hospital mortality was extremely rare, while morbidity rates were also low, though some differences emerged among the different treatments. Nonadjustable slings were associated with a slightly higher risk of acute urinary retention than AUS, but with a lower risk of surgical wound infections. The differences in the urinary retention rates are likely attributable to the postoperative swelling and the fact that the AUS is implanted in a deactivated state. Of note, adjustable slings had the lowest rate of acute urinary retention, with infection and sepsis rates below 1.0%.

Overall, the reported complication rates are consistent with those described in previous smaller series [26], [27]. These data underscore that all procedures are safe, but their perioperative profiles differ slightly, which may guide patient counseling and shared decision-making. Interestingly, within the AUS group, two-cuff devices were linked to slightly higher rates of acute urinary retention, without differences in infection, sepsis, or mortality. Thus, potential continence benefits of two-cuff systems may come at the cost of increased perioperative morbidity. This finding is in line with a study by Ahyai et al [28], which demonstrated a 5.7-fold higher explantation rate after two-cuff AUS. Similarly, Kretschmer et al [29] found two-cuff placement to be an independent predictor for AUS failure. Of note, LOS was procedure-dependent, with AUS patients staying a median of 6 d, compared with 5 d for both sling types. This difference may be explained by distinct perioperative antibiotic prophylaxis concepts applied to AUS and sling procedures in Germany, leading to longer intravenous antibiotics courses, therefore, prolonging the LOS [30].

The analysis on explantation rates offered important insights into device durability. In total, 5700 AUS explantations were performed, most often due to infection or mechanical failure, followed by urethral erosion. Other series have likewise shown that infection represents the most frequent indication for AUS explantation [31]. However, urethral erosion often leads to secondary infection, which may have been miscoded, thereby explaining why infection was the most common reason for explantation in our cohort. Overall, urethral erosion remains a major concern in AUS and remains the leading cause of explantation [32]. On the contrary, among 1435 explanted adjustable slings, infections (29%) and mechanical failure (28%) were less frequent, and nearly 40% were attributed to other causes. The latter is most likely attributable to insufficient continence outcomes and a subsequent conversion to AUS, which, however, can be performed without adversely affecting AUS results [33]. Furthermore, this more heterogeneous pattern may reflect differences in patient selection. Concomitant explantation with immediate reimplantation was recorded in 2629 AUS and 2531 sling cases. For AUS, nearly half of reimplantation were due to mechanical failure, while sling reimplantation were more often related to diverse causes, with mechanical failure accounting for only 6.1%. These findings suggest that AUS durability is most limited by infection and device wear, whereas sling outcomes may be influenced by a broader spectrum of complications.

Despite the large sample size of this study, several limitations must be acknowledged. First, our analysis relies on administrative data, which is primarily collected for reimbursement purposes rather than research. As a result, coding inaccuracies or misclassification of diagnoses and procedures cannot be entirely ruled out. Comorbidities may be underreported, as not all conditions are consistently documented. In the present analysis, mortality and sepsis rates were overall low; therefore, these outcomes were reported descriptively only and not in multivariable regression models. Detailed clinical data, such as the severity of incontinence, its cause, surgical history, intraoperative findings, surgeon experience, catheterization duration, and prior treatments, as well as device-specific coding, were unavailable. Additionally, there is no tracking of implants to provide data on implant survival up to explantation. The dataset also lacked postoperative functional outcomes, long-term performance data, and patient-reported satisfaction. While this analysis primarily focuses on perioperative data, the relatively high number of explantations in the cohort provides only indirect insight into long-term device performance. Therefore, the results should be interpreted cautiously, as perioperative safety does not necessarily imply long-term efficacy or durability. Lastly, since the analysis was limited to the German healthcare system, its applicability to other settings may be restricted.

## 5 Conclusion

In this nationwide analysis, AUS implantation remained as the predominant treatment for male SUI in Germany, with excellent in-hospital safety. Sling procedures, particularly adjustable systems, are increasingly used and provide comparable safety. Explantation patterns highlight infection and mechanical failure as key challenges for AUS, while sling outcomes were more heterogeneous. Overall, male SUI surgery in Germany is safe and commonly used, with AUS remaining the gold standard for severe cases. Sling procedures, particularly adjustable systems, are steadily gaining ground for selected patients. However, the absolute number of procedures may suggest that a substantial proportion of men with SUI remain untreated, underscoring the need for increased awareness, improved access to care, and ongoing innovation in this field.

  ***Author contributions***: Yannic Volz had full access to all the data in the study and takes responsibility for the integrity of the data and the accuracy of the data analysis.

  *Study concept and design*: Pyrgidis, Volz, Schulz, Stief.

*Acquisition of data*: Pyrgidis, Schulz, Hermans.

*Analysis and interpretation of data*: Pyrgidis, Volz.

*Drafting of the manuscript*: Pyrgidis, Volz.

*Critical revision of the manuscript for important intellectual content*: Bauer, Gozzi, Marcon, Weinhold, Kidess.

*Statistical analysis*: Pyrgidis, Volz.

*Obtaining funding*: None.

*Administrative, technical, or material support*: None.

*Supervision:* Stief.

*Other* (specify): None.

  ***Financial disclosures:*** Yannic Volz certifies that all conflicts of interest, including specific financial interests and relationships and affiliations relevant to the subject matter or materials discussed in the manuscript (eg, employment/affiliation, grants or funding, consultancies, honoraria, stock ownership or options, expert testimony, royalties, or patents filed, received, or pending), are the following: Y.V. receives speaker fees and words as an advisor for AMI GmbH, Feldkirch, Austria as well as Boston Medizintechnik GmbH, Düsseldorf, Germany.

  ***Funding/Support and role of the sponsor*:** None.

  ***Ethics statement:*** Written informed consent from the participants, as well as ethical approval, was not required for the present study in accordance with the national legislation and institutional requirements. All data are collected and maintained by the Federal Statistical Office (Wiesbaden, Germany) and were accessed under an approved agreement (LMU - 4710-2022).
