# Supplementary material for: Evolving Trends and Perioperative Outcomes of Surgical Treatment for Male Stress Urinary Incontinence: Results from the GRAND Study Register
Source: Eur Urol Open Sci. 2026 Jun 22;90:42–9. doi: 10.1016/j.euros.2026.06.001 (PMC13316223; doi:10.1016/j.euros.2026.06.001)
Supplement: Supplementary Data 1 [file mmc1.docx]

| **Characteristic** | **One-cuff AUS**, n = 11,770 | **Two-cuff AUS**, n = 3,501 |
| --- | --- | --- |
| **Age (years)** | 72 (67-76) | 71 (66-75) |
| **Obesity** | 776 (6.6%) | 225 (6.4%) |
| **Hypertension** | 6,677 (57%) | 1,909 (55%) |
| **Diabetes** | 2,195 (19%) | 613 (18%) |
| **Chronic kidney disease** | 730 (6.2%) | 270 (7.7%) |
| **Chronic heart failure** | 298 (2.5%) | 104 (3.0%) |
| **Prior radiation therapy** | 1,636 (14%) | 389 (11%) |
| **Age group** |  |  |
| <49 | 117 (1.0%) | 30 (0.9%) |
| 50-59 | 543 (4.6%) | 168 (4.8%) |
| 60-69 | 3,204 (27%) | 1,013 (29%) |
| 70-79 | 6,415 (55%) | 1,968 (56%) |
| >80 | 1,491 (13%) | 322 (9.2%) |

Supplementary Table 1: Baseline characteristics of one-cuff AUS and two-cuff AUS. Variables are presented as median with interquartile range or frequencies with proportions. AUS: artificial urinary sphincter.
